# Supplementary material for: Determination of the lipid composition of the GPI anchor
Source: PLoS One. 2021 Aug 13;16(8):e0256184. doi: 10.1371/journal.pone.0256184 (PMC8362999; doi:10.1371/journal.pone.0256184)
Supplement: S1 File — (PDF) [file pone.0256184.s001.pdf]

Determination of the lipid composition of the GPI anchor

Auxiliadora Aguilera-Romero †[1], Susana Sabido-Bozo †[1], Sergio Lopez[1], Alejandro Cortes-Gomez[1], Sofia Rodriguez-Gallardo[1], Ana Maria Perez-Linero[1], Isabelle Riezman[2], Howard Riezman[2], Manuel Muñiz[1]

[1]Departamento de Biología Celular, Facultad de Biología, Universidad de Sevilla e Instituto de Biomedicina de Sevilla (IBiS), Hospital Universitario Virgen del Rocío/CSIC/Universidad de Sevilla, 41012, Seville, Spain;

[2]NCCR Chemical Biology, Department of Biochemistry, University of Geneva, 1211, Geneva, Switzerland

Auxiliadora Aguilera-Romero †: Corresponding author. Email: auxi@us.es. †These authors contributed equally to this work.;
Susana Sabido-Bozo †: †These authors contributed equally to this work.
Manuel Muñiz: Corresponding author. E-mail: mmuniz@us.es

1 Works for me

Manuel Muniz

ABSTRACT

In eukaryotic cells, a subset of cell surface proteins is attached by the glycolipid glycosylphosphatidylinositol (GPI) to the external leaflet of the plasma membrane where they play important roles as enzymes, receptors, or adhesion molecules. Here we present an improved protocol for purification and mass spectrometry analysis of the lipid moiety of individual GPI-anchored proteins (GPI-APs) in yeast. The method involves the expression of a specific GPI-AP tagged with GFP, solubilization, immunoprecipitation, separation by electrophoresis, blotting onto PVDF, release, extraction of the GPI-lipid moiety, and analysis by mass spectrometry. By using this protocol, we could determine the precise GPI-lipid structure of the GPI-AP Gas1-GFP in a modified yeast strain. This protocol can be used to identify the lipid composition of the GPI-anchor of distinct GPI-APs from yeast to mammals and can be adapted to determine other types of protein lipidation.

THIS PROTOCOL ACCOMPANIES THE FOLLOWING PUBLICATION

S. Rodriguez-Gallardo et al., Ceramide chain length-dependent protein sorting into selective endoplasmic reticulum exit sites. Sci Adv 6, (2020). DOI: 10.1126/sciadv.aba8237

PROTOCOL INFO

Auxiliadora Aguilera-Romero †, Susana Sabido-Bozo †, Sergio Lopez, Alejandro Cortes-Gomez, Sofia Rodriguez-Gallardo, Ana Maria Perez-Linero, Isabelle Riezman, Howard Riezman, Manuel Muñiz . Determination of the lipid composition of the GPI anchor. **protocols.io**
https://protocols.io/view/determination-of-the-lipid-composition-of-the-gpi-bvban2ie

MANUSCRIPT CITATION please remember to cite the following publication along with this protocol

S. Rodriguez-Gallardo et al., Ceramide chain length-dependent protein sorting into selective endoplasmic reticulum exit sites. Sci Adv 6, (2020). DOI: 10.1126/sciadv.aba8237

KEYWORDS

GPI anchor, lipid moiety, GPI-anchored proteins, mass spectrometry

CREATED

May 26, 2021

LAST MODIFIED

May 28, 2021

PROTOCOL INTEGER ID

50242

MATERIALS TEXT

Recipes

Growth media
1. SD agar plates: synthetical minimal medium containing 2% glucose, 0.67% yeast nitrogen base, 0.5% ammonium sulfate and 2% bacteriological agar, supplemented with the appropriate nutritional requirements (0.012% except for adenine 0,03%) to complement auxotrophies.
2. SD medium: synthetical minimal medium containing 2% glucose, 0.67% yeast nitrogen base and 0.5% ammonium sulphate, supplemented with the appropriate nutritional requirements (0.012% except for adenine 0,03%) to complement auxotrophies.
3. YPD medium: rich medium containing 2% glucose, 1% yeast extract and 2% peptone, supplemented with 0.2% adenine and uracil.

Buffers
1. 1000x PI (Protease inhibitor cocktail): antipain 0.1%, leupeptin 0.1% and pepstatin 0.1% in DMSO
2. PMSF: 100mM phenylmethylsulphonyl fluoride in isopropanol
3. TNE: 50 mM Tris-HCl, pH 7.5, 150 mM NaCl, 5 mM EDTA, 1 mM PMSF, 1x PI
4. TNE-D 5%: TNE containing 5% (w/v) digitonin
5. TNE-D 1%: TNE containing 1% (w/v) digitonin
6. TNE-D 0.2%: TNE containing 0.2% (w/v) digitonin
7. Stock of Ammonium acetate 1M (MS grade) in water (MS grade)
8. Destaining solution: Isopropanol 20%, Acetic acid 10%
9. SB 2X (Sample buffer): 4% SDS, 20% glycerol, 10% 2-mercaptoethanol, 0.004% bromophenol blue and 0.125 M Tris HCl, pH 6.8

Equipment
1. Incubator shaker (Eppendorf, model M1299-0092)
2. Micropipettes (Gilson, Pipetman, models: P20, P200, P1000)
3. Spectrophotometer or microplate reader (any brand with 600 nm wavelength) or equipment to count cell density
4. Fastprep (MP Biomedicals, model: Fastprep-24).
5. Centrifuge (Thermo scientific, model: Heraeus Fresco 17; Eppendorf, model: centrifuge 5818R)
6. Orbital wheel (any brand)
7. Mini PROTEAN (Bio-Rad catalog number 1658005)
8. Power supply (Bio-Rad, catalog number: 1645052)
9. Amber glass vials (Agilent Part N. 5190-9063)
10. Hamilton syringe.
11. 96 well plate (Eppendorf twin.tec PCR 96 well plate)
12. Easy Pierce 20µm Foil (Thermo Scientific)
13. Triversa Nanomate (Advion, Ithaca, NY)
14. TSQ Vantage (ThermoFisher Scientific, Waltham, MA)

Materials
1. Budding yeast cells (W303) expressing Gas1-GFP
2. Toothpicks sterilized before use (any brand)
3. Yeast extract (Pronadisa catalog number: 1702.00)
4. Peptone (Panreac catalog number: A2210,0500)
5. Glucose (Panreac catalog number: 131341.0914)
6. Yeast nitrogen base without amino acids (Difco Laboratories, catalog number: 291940)
7. Nutritional supplements
   a. Uracil SIGMA catalog number: U0750
   b. L-Leucine SIGMA catalog number: L8000
   c. L-Tryptophan SIGMA catalog number: T0254
   d. Adenine SIGMA catalog number: A8626
   e. L-Histidine SIGMA catalog number: H8000
   f. L(+)-Lysine SIGMA catalog number: L5501
   g. L-Methionine SIGMA catalog number: M9625)
8. Agar (Oxoid catalog number: LP0011)
9. 50 ml Falcon tubes (any brand)
10. 1.5 ml screw-cap tubes (any brand)
11. Glass beads (SIGMA catalog number: G9268-500G)
12. Ultraclear 1.5 tubes (Axygen catalog number: MTC-175-L-C)
13. Digitonin (Panreac AppliChem catalog number: A1905,0005)
14. GFP-Trap Agarose beads (ChromoTek, catalog number: gta-20)
15. Antipain (SIGMA catalog number: A6191)
16. Leupeptin (SIGMA catalog number: L2023)
17. Pepstatin A (SIGMA catalog number: P4265)

18. DMSO (Sigma catalog number: 154938-100ML)
19. PMSF (Amresco catalog number: 0754-5G)
20. Isopropanol (Panreac catalog number: 131090.1211)
21. Tris (Amresco catalog number: 0497-1KG)
22. NaCl (Panreac catalog number: 131659.1211)
23. EDTA (Amresco catalog number: 0322-500G)
24. HCl 5mol/l (Panreac catalog number: 182109.1211)
25. Methanol (Panreac catalog number: 131091.1214)
26. Acetic Acid Glacial (Panreac catalog number: 141008.1211)
27. PVDF membranes (Thermo Scientific catalog number: 88520)
28. Amido Black Staining Solution 2X (Sigma A8181)
29. Methanol MS-LC (Fischer scientific catalog number: 15514065)
30. Sodium nitrite (Merck 106549)
31. NaOAc (Amresco 0602)
32. Chloroform HPLC (Merck catalog number: 650471)
33. Water, CHROMASOLV™ LC-MS (Fischer scientific catalog number: 15665350)
34. Ammonium Acetate (Merck catalog number 73594)

Yeast growth and culture

1 Transform the yeast strain with a centromeric plasmid expressing GFP-tagged Gas1p under control of its own promoter (pRS416-GAS1-GFP).

2 Pick up a colony and streak it on a SD agar plate with appropriate nutritional supplements (SC-URA) and incubate them at 24 °C for 2-3 days.

3 Inoculate transformed yeast cells in 3ml of SC-URA in a 12 ml sterile tube and growth them to the early-to-mid logarithmic phase at 24 °C with shaking at 250 rpm.

4 Dilute yeast cells into 800 ml of YPD medium and growth them to mid-log phase overnight at 30°C with shaking at 250 rpm. YPD medium is required for the correct expression of Gas1-GFP at the cell surface.

5 On the next day, harvest 600 x 10^7 cells by centrifugation at 3000 x g for 5 min, discard the supernatant, resuspend in 12 ml of TNE precooled at 4°C and distribute aliquots of 1 ml into 2 ml screw-cap tubes (12 tubes).

6 Centrifuge aliquots at 13,000 x g for 1 min, discard supernatant and freeze the pellet at -80 °C.

Immunoprecipitation of Gas1-GFP

7 Add 300 µl of glass beads and 1.5 ml of TNE to each cell pellet.

8 Break cells using Fast Prep (3 pulses of 30 s at 5 m/s).

9 Remove glass beads and cell debris by centrifugation at 1000 x g for 10 min at 4 °C.

10 Transfer 1 ml of supernatant into a 1.5 ml ultraclear tube (UC).

11 Centrifuge at 17,000 x g for 20 min at 4 °C.

12 Resuspend and combine all the pellets into a single 1.5 ml UC tube using 1 ml of TNE.

13 Add 250µl of TNE-D 5 % to obtain a solution with a final concentration of 1% digitonin. Mix with end-over-end rotation for 1 h at 4 °C.

14 Centrifuge the suspension at 17,000 x g for 20 min at 4 °C to remove insoluble material.

15 Transfer the supernatant to an UC tube with 100 µl GFP-Trap beads at (30 % slurry) and incubate overnight at 4 °C.

16 Next day, centrifuge at 5,000 x g for 30 s at 4 °C and remove supernatant.

17 Resuspend the beads pellet with 500 µl of TNE-D 1 % and transfer to a new UC tube.

18 Wash the original UC tube with 500 µl of TNE-D 1 % and combine the beads suspension with the first one.

19 Centrifuge at 5,000 x g for 30 s at 4 °C and remove the supernatant.

20 Add 750 µl TNE-D 0.2 %. Repeat step 19 and 20 twice more.

21 Centrifuge at 5,000 x g for 30 s at 4 °C and remove the supernatant. Centrifuge again to remove the remaining liquid and dry the beads using a white micropipette tip.

22 Add 40 µl of SB 2x.

23 Heat the samples at 95 °C for 10 min.

24 Vortex. Centrifuge maximum speed for 5 min at room temperature.

25 Load the supernatant onto a 7.5 % acrylamide gel, separate the proteins by SDS-PAGE gel electrophoresis and transfer to a PVDF membrane previously activated by methanol (Wet the PVDF membrane in 100 % methanol for a few seconds and then equilibrate the membrane in transfer buffer for a few minutes until it sinks).

26 After transfer, stain the PVDF membrane with Amido Black Staining Solution 2x for 1min.

27 Wash five times with the destaining solution for 2 min.

28 Cut out Gas1-GFP band with a new razor blade and transfer to an amber glass tube or screw-cap Eppendorf tube.

Lipid extraction from GPI anchor

29 Wash PVDF membrane strips slowly avoiding touching the membrane with 1ml of methanol (MS quality) four times.

30 Wash PVDF strips with 1ml of water (MS quality).

31 Incubate with a filtered mixture of 500 µl of 0.3 M NaOAc pH 4.0 buffer and 500 µl of freshly dissolved 1 M $NaNO_2$ for 3 h at 37 °C.

32 Remove the solution and wash the PVDF membrane strips with 1 ml of water MS-LC grade four times.

33 Dry and transfer the membrane to an amber tube. Flush the samples with N^2 and store at -80 °C.

Mass spectrometric analysis of IPC from Gas1-GFP

34 Extract the GPI-lipids from the PVDF strip with 75 µl of negative mode solvent (Chloroform/Methanol (1:2) + 5 mM Ammonium acetate).

35 Transfer the sample into a 96 well plate and seal with an Easy Pierce 20 µm Foil to avoid evaporation of the sample.

36 Infuse the sample on a TSQ Vantage using a Triversa Nanomate equipped with a HD_A chip with a gas pressure of 30 psi and a spray voltage of 1.2 kV.

Operate the TSQ Vantage with a spray voltage of 3 kV in negative-ion mode. The capillary temperature is set to 190 °C.

37

38 Use Multiple Reaction Monitoring (MRM) to obtain the signal intensity for each lipid molecular species (Fragmentation is performed with a collision energy optimized for each lipid class).

39 Read 3 times the MRM list (Table 1) on the TSQ Vantage to obtain three technical replicates for each biological replicate of the strain (It is advisable, to include at least, 3 biological replicates per strain) The MRM measurements are done with a width set to 1.0 in the Q3.

40 Calculate the signal intensity for each parent ion adding the intensities obtained in a range of ± 0.5 the m/z of the production. This can be done manually with Thermo Xcalibur software.

41 Finally, calculate the proportional signal for each lipid species compared to the sum of all the signals.
