# Supplementary material for: Determination of the lipid composition of the GPI anchor
Source: PLoS One. 2021 Aug 13;16(8):e0256184. doi: 10.1371/journal.pone.0256184 (PMC8362999; doi:10.1371/journal.pone.0256184)
Supplement: S1 Table — (PDF) [file pone.0256184.s002.pdf]

S1 Table: m/z of Precursor/product ion for multiple-reaction monitoring of sphingolipid species of the yeast *Saccharomyces cerevisiae* used in this study

Lipid species	Precursor ion (Q1)	Product ion (Q3)
IPC-A d32:0	752,508	240,58-241,51
IPC-A d34:0	780,539	240,58-241,51
IPC-A d36:0	808,57	240,58-241,51
IPC-A d38:0	836,602	240,58-241,51
IPC-A d40:0	864,633	240,58-241,51
IPC-A d42:0	892,664	240,58-241,51
IPC-A d44:0	920,696	240,58-241,51
IPC-A d46:0	948,727	240,58-241,51
IPC-A d48:0	976,758	240,58-241,51
IPC-B d/t32:0	768,503	240,58-241,51
IPC-B d/t34:0	796,534	240,58-241,51
IPC-B d/t36:0	824,565	240,58-241,51
IPC-B d/t38:0	852,597	240,58-241,51
IPC-B d/t40:0	880,628	240,58-241,51
IPC-B d/t42:0	908,659	240,58-241,51
IPC-B d/t44:0	936,69	240,58-241,51
IPC-B d/t46:0	964,722	240,58-241,51
IPC-B d/t48:0	992,753	240,58-241,51
IPC-C t32:0	784,498	240,58-241,51
IPC-C t34:0	812,529	240,58-241,51
IPC-C t36:0	840,56	240,58-241,51
IPC-C t38:0	868,591	240,58-241,51
IPC-C t40:0	896,623	240,58-241,51
IPC-C t42:0	924,654	240,58-241,51
IPC-C t44:0	952,685	240,58-241,51
IPC-C t46:0	980,717	240,58-241,51
IPC-C t48:0	1008,748	240,58-241,51
IPC-D t32:0	800,493	240,58-241,51
IPC-D t34:0	828,524	240,58-241,51
IPC-D t36:0	856,555	240,58-241,51
IPC-D t38:0	884,586	240,58-241,51
IPC-D t40:0	912,618	240,58-241,51
IPC-D t42:0	940,649	240,58-241,51
IPC-D t44:0	968,68	240,58-241,51
IPC-D t46:0	996,712	240,58-241,51
IPC-D t48:0	1024,743	240,58-241,51
M(IP)2C-A d32:0	577,786	240,58-241,51
M(IP)2C-A d34:0	591,802	240,58-241,51
M(IP)2C-A d36:0	605,817	240,58-241,51
M(IP)2C-A d38:0	619,833	240,58-241,51
M(IP)2C-A d40:0	633,849	240,58-241,51
M(IP)2C-A d42:0	647,864	240,58-241,51
M(IP)2C-A d44:0	661,88	240,58-241,51
M(IP)2C-A d46:0	675,895	240,58-241,51
M(IP)2C-A d48:0	689,911	240,58-241,51
M(IP)2C-B d/t32:0	585,783	240,58-241,51
M(IP)2C-B d/t34:0	599,799	240,58-241,51
M(IP)2C-B d/t36:0	613,815	240,58-241,51

M(IP)2C-B d/t38:0	627,83	240,58-241,51
M(IP)2C-B d/t40:0	641,846	240,58-241,51
M(IP)2C-B d/t42:0	655,862	240,58-241,51
M(IP)2C-B d/t44:0	669,877	240,58-241,51
M(IP)2C-B d/t46:0	683,893	240,58-241,51
M(IP)2C-B d/t48:0	697,909	240,58-241,51
M(IP)2C-C t32:0	593,781	240,58-241,51
M(IP)2C-C t34:0	607,797	240,58-241,51
M(IP)2C-C t36:0	621,812	240,58-241,51
M(IP)2C-C t38:0	635,828	240,58-241,51
M(IP)2C-C t40:0	649,843	240,58-241,51
M(IP)2C-C t42:0	663,859	240,58-241,51
M(IP)2C-C t44:0	677,875	240,58-241,51
M(IP)2C-C t46:0	691,89	240,58-241,51
M(IP)2C-C t48:0	705,906	240,58-241,51
M(IP)2C-D t32:0	601,778	240,58-241,51
M(IP)2C-D t34:0	615,794	240,58-241,51
M(IP)2C-D t36:0	629,81	240,58-241,51
M(IP)2C-D t38:0	643,825	240,58-241,51
M(IP)2C-D t40:0	657,841	240,58-241,51
M(IP)2C-D t42:0	671,857	240,58-241,51
M(IP)2C-D t44:0	685,872	240,58-241,51
M(IP)2C-D t46:0	699,888	240,58-241,51
M(IP)2C-D t48:0	713,904	240,58-241,51
MIPC-A d32:0	914,561	420,64-421,57
MIPC-A d34:0	942,592	420,64-421,57
MIPC-A d36:0	970,623	420,64-421,57
MIPC-A d38:0	998,654	420,64-421,57
MIPC-A d40:0	1026,686	420,64-421,57
MIPC-A d42:0	1054,717	420,64-421,57
MIPC-A d44:0	1082,748	420,64-421,57
MIPC-A d46:0	1110,78	420,64-421,57
MIPC-A d48:0	1138,811	420,64-421,57
MIPC-B d/t32:0	930,555	420,64-421,57
MIPC-B d/t34:0	958,587	420,64-421,57
MIPC-B d/t36:0	986,618	420,64-421,57
MIPC-B d/t38:0	1014,649	420,64-421,57
MIPC-B d/t40:0	1042,681	420,64-421,57
MIPC-B d/t42:0	1070,712	420,64-421,57
MIPC-B d/t44:0	1098,743	420,64-421,57
MIPC-B d/t46:0	1126,775	420,64-421,57
MIPC-B d/t48:0	1154,806	420,64-421,57
MIPC-C t32:0	946,55	420,64-421,57
MIPC-C t34:0	974,582	420,64-421,57
MIPC-C t36:0	1002,613	420,64-421,57
MIPC-C t38:0	1030,644	420,64-421,57
MIPC-C t40:0	1058,676	420,64-421,57
MIPC-C t42:0	1086,707	420,64-421,57
MIPC-C t44:0	1114,738	420,64-421,57
MIPC-C t46:0	1142,769	420,64-421,57

MIPC-C t48:0	1170,801	420,64-421,57
MIPC-D t32:0	962,545	420,64-421,57
MIPC-D t34:0	990,577	420,64-421,57
MIPC-D t36:0	1018,608	420,64-421,57
MIPC-D t38:0	1046,639	420,64-421,57
MIPC-D t40:0	1074,671	420,64-421,57
MIPC-D t42:0	1102,702	420,64-421,57
MIPC-D t44:0	1130,733	420,64-421,57
MIPC-D t46:0	1158,764	420,64-421,57
