# Supplementary material for: Determination of the lipid composition of the GPI anchor
Source: PLoS One. 2021 Aug 13;16(8):e0256184. doi: 10.1371/journal.pone.0256184 (PMC8362999; doi:10.1371/journal.pone.0256184)
Supplement: S2 Table — (PDF) [file pone.0256184.s003.pdf]

S2 Table: *m/z* of Precursor/product ion for multiple-reaction monitoring of phosphatidylinositol lipid species from the yeast *Saccharomyces cerevisiae*

	Precursor ion (Q1)	Product ion (Q3)
LysoPI14:0	543,25703	240,58-241,51
LysoPI14:1	541,24138	240,58-241,51
LysoPI16:0	571,28833	240,58-241,51
LysoPI16:1	569,27268	240,58-241,51
LysoPI18:0	599,31963	240,58-241,51
LysoPI18:1	597,30398	240,58-241,51
LysoPI20:0	627,35093	240,58-241,51
LysoPI20:1	625,33528	240,58-241,51
LysoPI22:0	655,38222	240,58-241,51
LysoPI22:1	653,36657	240,58-241,51
LysoPI24:0	683,41352	240,58-241,51
LysoPI24:1	681,39787	240,58-241,51
LysoPI26:0	711,44482	240,58-241,51
LysoPI26:1	709,42917	240,58-241,51
PI26:0	725,42409	240,58-241,51
PI26:1	723,40844	240,58-241,51
PI26:2	721,39279	240,58-241,51
PI28:0	753,45538	240,58-241,51
PI28:1	751,43974	240,58-241,51
PI28:2	749,42409	240,58-241,51
PI30:0	781,48668	240,58-241,51
PI30:1	779,47103	240,58-241,51
PI30:2	777,45538	240,58-241,51
PI31:1	793,486563	240,58-241,51
PI32:0	809,51798	240,58-241,51
PI32:1	807,50233	240,58-241,51
PI32:2	805,48668	240,58-241,51
PI34:0	837,54928	240,58-241,51
PI34:1	835,53363	240,58-241,51
PI34:2	833,51798	240,58-241,51
PI36:0	865,58058	240,58-241,51
PI36:1	863,56493	240,58-241,51
PI36:2	861,54928	240,58-241,51
PI38:0	893,61188	240,58-241,51
PI38:1	891,59623	240,58-241,51
PI38:2	889,58058	240,58-241,51
PI40:0	921,64318	240,58-241,51
PI40:1	919,62753	240,58-241,51
PI40:2	917,61188	240,58-241,51
PI42:0	949,67447	240,58-241,51
PI42:1	947,65882	240,58-241,51
PI42:2	945,64318	240,58-241,51
PI44:0	977,70577	240,58-241,51
PI44:1	975,69012	240,58-241,51
PI44:2	973,67447	240,58-241,51
PI46:0	1005,73707	240,58-241,51
PI46:1	1003,72142	240,58-241,51

PI46:2	1001,70577	240,58-241,51
PI48:0	1033,76837	240,58-241,51
PI48:1	1031,75272	240,58-241,51
PI48:2	1029,73707	240,58-241,51
