# Supplementary material for: Determination of the lipid composition of the GPI anchor
Source: PLoS One. 2021 Aug 13;16(8):e0256184. doi: 10.1371/journal.pone.0256184 (PMC8362999; doi:10.1371/journal.pone.0256184)
Supplement: Attachment — Submitted filename: Aguilera-romero_response to reviewers.pdf [file pone.0256184.s004.pdf]

Point by Point response

Reviewer #1:

The protocol article written by Aguilera-Romero et al described the lipid analysis of GPI-anchored proteins. The authors showed an analytical method of inositol phosphoceramide structures on GFP-tagged Gas1 proteins expressed in Saccharomyces cerevisiae. It would be useful for readers who are interested in lipid species of GPI-anchors and their changes by mutations in genes. The reviewer recommends the publication after the appropriate revision.

We thank the reviewer for the very positive and helpful comments on our manuscript, and we hope to have answered all concerns raised.

1) In the abstract, the authors wrote "an improved protocol". Please describe more clearly what is improved compared to the previous methods (Fontaine et al. (2003) Glycobiology 13(3):169-77; Yoko-o et al. (2013) Mol. Microbiol. 88(1):140-55). Compared to the old methods, purification of PI using silica column was omitted. Is it because of changing the detection method using MRM in the analysis by ESI-MS/MS?

Response: As pointed out by the reviewer, the adjective "improved" is not appropriate because our protocol is a compilation of methods used in Fontaine et al. (2003) Glycobiology 13(3):169-77, Yoko-o et al. (2013) Mol. Microbiol. 88(1):140-55 and Mehlert et al. (2009) Glycoconj J. 26(8):915-921. Therefore, we remove this adjective from the abstract. Following the method used by Mehlert et al. (2009) we decided to directly infuse the sample on the MS/MS without a previous purification by silica column. Thanks to the use of a targeted approach, MRM, we could clearly identify the lipid specie of the GPI anchor despite of background noise. This simplifies the method allowing more routine studies. However, for a more explorative approach we consider the protocol described in Yoko-o et al. (2013) to be more suitable. The text is now corrected as suggested by the reviewer.

2) The authors listed the ions for MRM of lipids in Table 1. In the list, not only IPC species, but also MIPC and M(IP)2C species are listed. MIPC and M(IP)2C should be removed from the list since these lipid species are not observed in the GPI lipids. Instead, is it possible to add diacylglycerol-type PI species?

Response: In Rodriguez-Gallardo et al. (2020) (Sci Adv 6(50):eaba8237) we studied the role of very long (C26) chain membrane ceramides in protein sorting from the ER. For this purpose, we used a modified yeast strain GhLag1, that produces cellular membranes with only long (C18) rather than very long (C26) chain ceramides (Epstein et al. (2012) Mol Microbiol 84, 1018). Gas1, used in this work as a reporter, is a GPI-AP that has a C26 ceramide lipid in its GPI-anchor (Yoko-o et al. (2013) Mol. Microbiol. 88(1):140-55). Since the strain GhLag1 mainly produces C18 ceramides we wanted to determine whether Gas1 incorporates a C18 or a C26 ceramide in its GPI anchor. To achieve this,

we developed the present method and applied a MRM list used routinely in our lab to define the sphingolipid composition of yeast strains, which contains IPC, MIPC and MIP2C lipid species. We agree with the reviewer comment that MIPC and MIP2C species are not required for our purpose, but we want to show the exact method used in the published paper that corresponds with the figure provided. Additionally, we think that the suggestion provided by the reviewer could be of interest to determine the lipid of the GPI-anchor therefore we provide an additional S2 table that contains a diacylglycerol-type PI MRM list.

Reviewer #2:
This protocol, presented here at the detail of a laboratory protocol, provides a highly standard series of methods to identify the lipid attached to an immunoaffinity tagged GPI-anchored protein. The force of these methods to identify lipid attachments to GPI-proteins is shown fully in their recent publication in Science Advances 2020;6(49). Developed in yeast, such is the importance of identifying lipid modification in all species that I am confident this protocol will be used widely in the future.
We thank the reviewer for the appreciation of our manuscript.

Reviewer #3:
The authors provide an excellent job of summarizing the significance and relevance of improving the protocol for detection of GPI-AP in yeast. This includes a detailed explanation of the roles of GPI and the distinctions between that of yeast and mammalian. Additionally, a comprehensive description of the rational for use of mass spectrometry-based methods versus the use of traditional methods. The authors' observations regarding the significance of using the ceramide acyl chain length for the ER protein. There are a few clarifications that I am looking for:

We thank the reviewer for the useful comments, and we hope to have answered the questions with the following.

1) How many samples were used for each the wild type and GhLag1, and just one blank were used? Were any biological or technical replicated used?

Response: To obtain the results published in Rodriguez-Gallardo et al. (2020) (Sci Adv 6(50):eaba8237) we performed five experiments, each of them with its blank, Wild-type and Ghlag1 sample. Thus, we obtained a total of 5 biological replicates by sample. For each of them each transition of the MRM list was read three times (three technical replicates). We have added this information to the manuscript.

2) Which instrument/columns were used for MS/MS?
The mass spectrometer used in our study is a TSQ Vantage and samples were infused on it using a Triversa Nanomate. We have added this information to the manuscript.
